# Cost‐Effectiveness of a Workplace‐Based Hypertension Management Program in Real‐World Practice in the Kailuan Study

**DOI:** 10.1161/JAHA.123.031578

**Published:** 2024-04-02

**Authors:** Yan‐Feng Zhou, Shuohua Chen, Jun‐Xiang Chen, Simiao Chen, Guodong Wang, Xiong‐Fei Pan, Shouling Wu, An Pan

**Affiliations:** ^1^ Department of Social Medicine, School of Public Health Guangxi Medical University Nanning China; ^2^ Department of Cardiology, Kailuan General Hospital North China University of Science and Technology Tangshan China; ^3^ Department of Epidemiology and Biostatistics, School of Public Health, Tongji Medical College Huazhong University of Science and Technology Wuhan China; ^4^ Chinese Academy of Medical Sciences and Peking Union Medical College Beijing China; ^5^ Heidelberg Institute of Global Health, Faculty of Medicine and University Hospital Heidelberg University Heidelberg Germany; ^6^ Key Laboratory of Birth Defects and Related Diseases of Women and Children (Sichuan University), Ministry of Education, West China Second University Hospital, Sichuan University Shuangliu Institute of Women’s and Children’s Health, Shuangliu Maternal and Child Health Hospital Chengdu China

**Keywords:** blood pressure, economic evaluation, health consultation, hypertension, workplace‐based, Hypertension, Cost-Effectiveness

## Abstract

**Background:**

In 2009, a workplace‐based hypertension management program was launched among men with hypertension in the Kailuan study. This program involved monitoring blood pressure semimonthly, providing free antihypertensive medications, and offering personalized health consultations. However, the cost‐effectiveness of this program remains unclear.

**Methods and Results:**

This analysis included 12 240 participants, with 6120 in each of the management and control groups. Using a microsimulation model derived from 10‐year follow‐up data, we estimated costs, quality‐adjusted life years (QALYs), life‐years, and incremental cost‐effectiveness ratios (ICERs) for workplace‐based management compared with routine care in both the study period and over a lifetime. Analyses are conducted from the societal perspective. Over the 10‐year follow‐up, patients in the management group experienced an average gain of 0.06 QALYs with associated incremental costs of $633.17 (4366.85 RMB). Projecting over a lifetime, the management group was estimated to increase by 0.88 QALYs or 0.92 life‐years compared with the control group, with an incremental cost of $1638.64 (11 301.37 RMB). This results in an incremental cost‐effectiveness ratio of $1855.47 per QALY gained and $1780.27 per life‐year gained, respectively, when comparing workplace‐based management with routine care. In probabilistic sensitivity analyses, with a threshold willingness‐to‐pay of $30 765 per QALY (3 times 2019 gross domestic product per capita), the management group showed a 100% likelihood of being cost‐effective in 10 000 samples.

**Conclusions:**

Workplace‐based management, compared with routine care for Chinese men with hypertension, could be cost‐effective both during the study period and over a lifetime, and might be considered in working populations in China and elsewhere.

**Nonstandard Abbreviations and Acronyms**
ICER: incremental cost‐effectiveness ratio
QALY: quality‐adjusted life year

**Clinical Perspective**

**What Is New?**

- Previous studies have indicated that workplace‐based hypertension management programs are effective in improving hypertension control; however, there is a lack of robust evidence regarding cost‐effectiveness.
- This study is the first investigation into the cost‐effectiveness of workplace‐based hypertension management programs among the Chinese population.

**What Are the Clinical Implications?**

- Our results showed that a hypertension management program in the workplace could be cost‐effective compared with routine care for Chinese men with hypertension, which might provide important evidence for policy makers and practitioners to develop cost‐effective workplace‐based hypertension strategies.

Hypertension is a preventable risk factor for global cardiovascular disease (CVD) and premature deaths.^1^ Despite advances in treatments and availability of low‐cost antihypertensive medications, hypertension control remains low in low‐ and middle‐income countries.^2^ A recent cross‐sectional study conducted in 44 low‐ and middle‐income countries, involving 1.1 million adults, revealed that merely 10% of individuals with hypertension had their blood pressure (BP) controlled below 140/90 mm Hg.^3^ In a screening study involving 1.7 million Chinese adults, only 45% were aware of their hypertension, 30% were treated, and merely 7.2% achieved BP control.^4^ Therefore, implementing multicomponent, cost‐effective, and sustainable hypertension prevention and control programs should be a key public health agenda for these countries.

We recently reported the results of a workplace‐based hypertension management program among male patients with hypertension in real‐world practice, a multicomponent strategy that included regular BP measurements, free antihypertensive medications, and personalized health consultations undertaken by community health workers, against routine care.^5^ We found that both systolic and diastolic BP were reduced in the management group compared with the control group at 10 years (−7.83 mm Hg and −4.72 mm Hg, respectively), showing that a workplace‐based hypertension management program held promise for achieving long‐term BP control. Consistent results were also reported in previous studies of workplace‐based hypertension management programs despite different effect sizes.^6^, ^7^, ^8^, ^9^, ^10^, ^11^ However, neither the cost nor the cost‐effectiveness of workplace‐based hypertension management programs was reported, although they are essential for policy makers and health planners who are making decisions on whether scarce health care system resources should be allocated to such programs or other priorities.

From a societal perspective, this study aimed to analyze the expenses linked to the workplace‐based hypertension management program and compare its incremental cost‐effectiveness with routine care.

## Methods

### Data Access

The underlying data for this article can be obtained from the corresponding author upon reasonable request.

### Model Population

The Kailuan study introduced a workplace‐based hypertension management program in 2009 that has lasted for 10 years among men with hypertension aged 18 to 60 years with a mean (SD) age being 45.4 (7.2) years. The comprehensive study design of the program was reported previously.^5^ Briefly, a general workplace wellness program was applied for all employees by community health workers and health professionals or researchers, following guidelines from the American Heart Association^12^ and the Chinese Hypertension Management.^13^ This program involved providing free health checkups every 2 years and providing health education sessions or activities at least twice a year.

Apart from the general workplace wellness program, the hypertension management program also involved monitoring BP semimonthly in community health centers, providing free antihypertensive medications, and offering personalized health consultations for male patients with hypertension. The community health workers underwent interactive training to become case managers for patients, coordinating interventions and supporting patient care, as well as helping schedule physician appointments. They received retraining every 2 years and were required to participate in continuous medical education at least once a year, which ensured that they were constantly updated on hypertension treatment or insurance reimbursement policies over time.

Participants in the control group were provided with the general workplace wellness program, but they did not participate in the hypertension management program and received only routine care for hypertension. While they did not receive free medication, they were enrolled in the Urban Employee Basic Medical Insurance scheme.^5^ In the control group, health insurance could partially cover medication expenses, the same as in the management group.

Eligible patients were invited to participate in the hypertension management program, with those who agreed to constitute the management group, and the remaining patients comprised the control group. To reduce the possibility of selection bias, a propensity‐matched analysis was used to choose participants for both the management and control groups, given the nonrandomized nature of program enrollment (Data S1). Initially, 17 724 men with hypertension aged 18 to 60 years were deemed eligible for the program, with 6400 patients ultimately participating (Figure S1).^5^ Propensity score matching resulted in 6120 participants each for both the management and control groups, ensuring a well‐balanced distribution of characteristics between the 2 groups (Table S1). Participants were followed until death or December 31, 2019.

This study received approval from the ethics committee of Kailuan General Hospital (Approval No. 2006‐05). Each participant provided written consent after being informed about the study details.

### Study Design

Following the guidelines and checklist items outlined in the Consolidated Health Economic Evaluation Reporting Standards.^14^ We conducted the cost‐effectiveness analysis both during the study period and over a lifetime. Throughout the study period, a cost‐effectiveness analysis was conducted using 10‐year follow‐up data on survival, CVD events, utilities, and costs from the hypertension management program. For the lifetime analysis, a microsimulation model was performed using data from the Kailuan population. This approach helped evaluate the lifetime costs, quality‐adjusted life years (QALYs), life years, and incremental cost‐effectiveness ratio (ICER) of workplace‐based management versus routine care from a societal perspective. This microsimulation simulated a 55‐year time horizon, encompassing a period in which the majority of individuals in the model would have died or reached the age of 100. The ICER that was <3 times the per‐capita gross domestic product (GDP) was deemed cost‐effective.^15^ The GDP per capita for China in 2019 was $10 255 (70 724.6 RMB) ($1.00=6.8968 RMB in 2019).^16^ The TreeAge Pro Suite 2022 (TreeAge Software, Inc., Williamstown, MA) was used for all analyses.

### Model Overview

**Figure 1:**
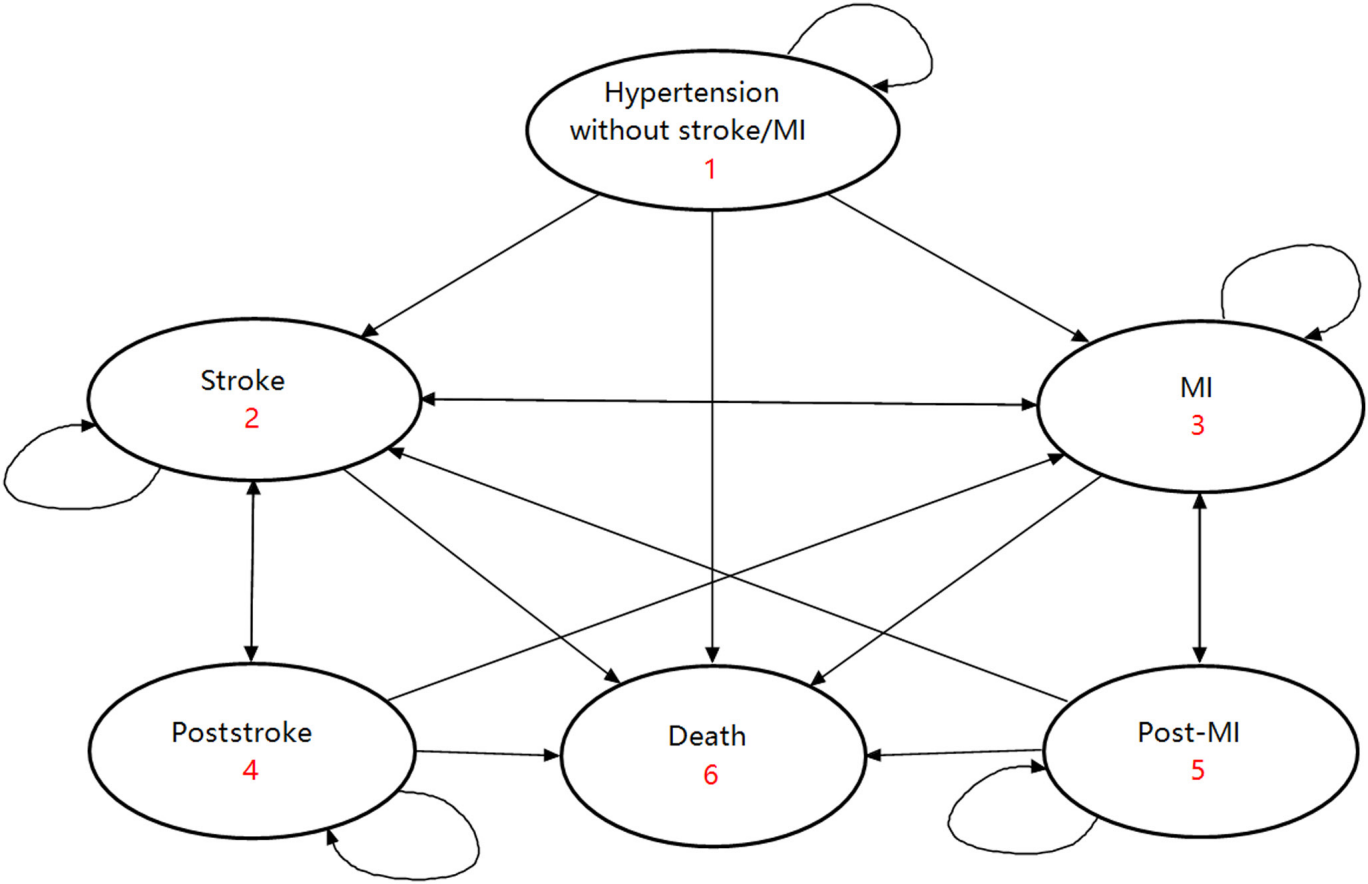
Markov state transition model for hypertension. MI indicates myocardial infarction.

To simulate hypertension progression, we constructed a Markov microsimulation model drawing from models published.^17^ Patients were assigned to 2 strategies, one adopting the hypertension management program (management group) and the other receiving routine care (control group) (Figure 1). Patients started in the model with hypertension, which was defined as systolic/diastolic BP ≥140/90 mm Hg, clinical diagnosis of hypertension, or receipt of antihypertensive medications. Patients stayed in their health state if they did not experience a stroke or myocardial infarction (MI) or died. Patients with a stroke or MI might move into a chronic health state (poststroke/post‐MI), experience a recurrent event, encounter additional complications, or die directly. The model cycled annually with a half‐cycle correction applied.

### Transition Probability

Annual probabilities of the first stroke originated from the model population in the Kailuan study on the basis of outcomes from claims databases or registries. We used a Weibull distribution to model patient survival in the control group, obtaining hypothetical parameter scale (λ) and a shape (γ). Transition probabilities for the management group were estimated by adjusting the hazard ratio (HR; 0.86) in the control group from the Kailuan study. Annual probabilities of the first MI were also obtained from the Kailuan study. Given the absence of a statistically significant difference in MI risk (*P*=0.12) between the management and control groups, we assumed equivalent annual MI probabilities across both groups. Annual probabilities of subsequent CVD events, including recurrent CVD, stroke incidence after MI/post‐MI, and MI incidence after stroke/poststroke, were obtained from other studies due to the unavailability of primary data in the Kailuan study.^18^, ^19^, ^20^, ^21^, ^22^, ^23^

Non‐CVD mortality rates in the control group were sourced from Chinese life tables corresponding to various age brackets derived from the sixth nationwide census.^24^ Mortality rates in the management group were estimated by adjusting the HR (0.74) for the control group from the Kailuan study. CVD mortality rates in the control group in the first year were obtained from the Kailuan study. Stroke mortality rate in the management group was estimated by adjusting the HR (0.68) for the control group. Mortality rates after recurrent CVD were obtained from other studies as data from the Kailuan study were not available.^20^ Since there were no statistical differences observed in the MI mortality rate and mortality rates after recurrent CVD (*P*>0.1), we assumed that patients had an equal likelihood of death following either an initial MI or recurrent CVD event. Event rates are summarized in Table 1.^25^

**Table 1:** Selected Input Value for the Cost‐Effectiveness Model

Parameters	Estimate (SD or range)	Distribution
Age, y, mean (SD)	45.4 (7.2)	NA
Rates and probabilities		
Morbidity rates, annual		
Primary stroke^5^	λ=0.00008, γ=1.805	Weibull
1‐year recurrent stroke,^18^ %	18	β
Long‐term recurrent stroke,^19^, ^20^ %	5.5 (2–9)*	β
MI incidence after stroke,^21^ %	0.4	β
MI incidence after poststroke,^21^ %	0.4	β
Primary MI,^5^ %	0.15	β
One‐year recurrent MI,^22^ %	3.2	β
Long‐term recurrent MI,^23^ %	1.2	β
Stroke incidence after MI,^23^ %	0.4	β
Stroke incidence after post‐MI,^23^ %	0.4	β
Mortality rates, annual		
Non‐CVD related death,^24^ %	0.07–50.728 (age and sex‐dependent)	Life table
After stroke,^5^ %	3.86	β
After poststroke,^20^ %	3	β
After MI, ^5^ %	3.62	β
After post‐MI, ^20^ %	1.5	β
Effect of hypertension management		
HR for risk of stroke^5^	0.86 (0.74–0.99)*	β
HR for non‐CVD death^5^	0.74 (0.60–0.92)*	β
HR for stroke death^5^	0.68 (0.56–0.83)*	β
Cost ($, per participant)†		
Annual cost for hypertension screening or checkup‡	28.04 (26.64–29.44)*	γ
Annual antihypertensive drug costs of 1.0 standard dose^25^	88.92 (51.24–266.76)*	γ
Annual intervention cost‡	131.42 (102.02–164.14)*	γ
Office rent and maintenance	30.33 (23.54–37.88)	γ
Equipment	12.05 (10.63–12.47)	γ
Platform development and maintenance	6.13 (6.09–6.16)	γ
Health care provider and administrative staff	62.99 (46.96–82.91)	γ
Training workers	0.30 (0.25–0.36)	γ
Management activities	19.29 (14.24–23.99)	γ
Training material and supplies	0.33 (0.31–0.37)	γ
Annual productivity loss‡	38.61 (12.87–51.49)* in the management group, 76.32 (25.44–101.76)* in the control group	γ
Annual cost for stroke^16^, ^17^	3170.30 (1002.35–9431.43)* for the first year, 1488.43 (314.38–4465.29)* for the subsequent years	γ
Annual cost for MI^16^, ^17^	4595.93 (1350.55–13570.66)* for the first year, 417.82 (137.07–1233.69)* for the subsequent years	γ
Quality‐of‐life weights (health utilities)		
Hypertension^17^	0.90 (0.79–0.95)*	β
Stroke^17^	0.63 (0.26–0.89)*; post 0.65 (0.46–0.82)*	β
MI^17^	0.76 (0.50–0.89)*; post 0.88 (0.67–0.94)*	β
Death	0	/
Discount rate, %	5 (0–8)*	β

CVD indicates cardiovascular disease; HR, hazard ratio; MI, myocardial infarction; and NA, not applicable.

* The values inside the parentheses represent ranges for sensitivity analyses.

^†^ The costs were inflated to the 2019 price level using the average rate of inflation in China from 2009 to 2019 and converted to US dollars ($1.00=6.8968 RMB).

^‡^ Data are from the Kailuan cohort.

### Costs

We conducted a retrospective cost analysis to assess the expenses associated with implementing the hypertension management program. The average annual cost was estimated for each participant from his enrollment date until December 31, 2019. The expenses encompassed hypertension screening or checkup expenses, management expenditures, antihypertensive medication costs, productivity losses, and expenses linked to stroke and MI. The hypertension screening or checkup costs were paid by the Kailuan group, whereas the management costs were deducted from the employee's salary by the company in the form of a project contract system. We averaged these costs from 2009 to 2019 with adjustments for inflation.

Particularly, hypertension screening or checkup costs included costs related to basic anthropometric measurement and laboratory examinations during the biennial checkup, which can be obtained from the financial expenditure of the Kailuan group. Management costs included costs for office rent and maintenance, equipment, platform development and maintenance, training, personnel, management activities, and office materials and supplies. We allocated an administrative overhead of 30% to the management expenses to accommodate office rental and maintenance expenses.^26^ Equipment expenditure included a 1‐time construction expenditure and medical equipment expenditure. Platform development and maintenance costs included the development and routine maintenance costs of the hypertension management platform. Training expenses included costs for trainers, training materials, curriculum development, transportation, and other operational needs. Personnel costs included salaries and fringe benefits of administrators and community health workers. Costs of equipment expenditure, platform development and maintenance, training, and personnel were obtained by a survey of the community health centers where the management program was conducted. Costs of management activities included costs for regular BP measurements, free antihypertensive medications, and personalized health consultations, which were obtained from the project contract system. Due to financial constraints, patients in the management group were given free access to 4 common antihypertensive medications (spironolactone, captopril, nitrendipine, and hydrochlorothiazide). Other antihypertensive medications could be obtained via visits to hospitals or pharmacies, with partial reimbursement through health insurance for medication costs.^13^ Although participants in the control group were not provided with free antihypertensive medications, participants were enrolled in the Urban Employee Basic Medical Insurance scheme and provided coverage,^5^ and medication expenses could be partially reimbursed by health insurance. Hence, the variations in the expenses for antihypertensive treatment between the 2 groups were minimal. As reported previously, the average systolic BP reduction was 9 mm Hg with a standard dose of drug treatment.^27^ To achieve an 8 mm Hg reduction in systolic BP in our study, we assumed a 1.0 standard dose of antihypertensive drug should be provided for patients, which was consistent with recommendations from the Chinese Hypertension Guidelines.^13^ We determined the average annual cost of antihypertensive drugs per standard dose by analyzing the yearly expenses and prescription patterns of 62 antihypertensive medications.^25^

Data on productivity losses were derived from the Social Security System, which tracked individuals' employment status and retirement decisions throughout the study. Other types of productivity loss, such as sickness absenteeism, were not included in this study because of a lack of relevant information. According to the provisional regulations of the State Council in China, the retirement age was 55 years for men who had engaged in underground work, at high altitudes, in high temperatures, or in particularly strenuous physical labor.^28^ Early retirement due to health issues can be characterized as leaving the workforce before the statutory retirement age due to health‐related factors. To determine the duration of early retirement, we computed the difference between the legal retirement age and the actual retirement age. Subsequently, productivity loss was determined by multiplying the duration of early retirement by the average wage. χ^2^ tests were used to determine whether there was a significant difference between the 2 groups in terms of the likelihood of early retirement. Two‐sided *P*<0.05 was considered as statistical significance. A lower estimate of productivity loss was calculated on the basis of one third of the average and a higher estimate on four thirds of the average wage (Tables S2 and S3).

The costs associated with CVD were derived from the electronic medical records in the Kailuan study and cross‐referenced with costs from the *China Health Statistics Yearbook 2019* ^16^ and the literature published.^17^ All costs were inflated to the 2019 price level on the basis of China's average inflation rate and reported in US dollars, with an annual discount rate of 5% (Table 1).

### QALY and Health Utilities

Utility values were derived from published literature, using the European Quality of Life‐5 Dimensions Questionnaire (Table 1).^5^ QALYs were computed by multiplying the duration spent in a specific health state by its corresponding utility value. QALYs were discounted annually at a rate of 5%.

### Main Analysis

The estimated model parameters, together with the assumed costs and utilities described above, were used to compute the ICER between the management group and control group, both during the study period and over the lifetime horizon.

### Sensitivity and Scenario Analyses

The variability surrounding the impact of model parameters on cost‐effectiveness was assessed through scenario and sensitivity analyses. In particular, scenario analyses were performed to examine how alternative model assumptions and parameter values affect the sensitivity of the cost‐effectiveness findings. For instance, we simulated that the management program has no benefit beyond year 10. Furthermore, given that the average age of the study population was 45.4 years and the retirement age was set at 55 years, productivity losses exclusively accounted for the initial 10 years of the microsimulation in an alternative scenario analysis.

One‐way and probabilistic sensitivity analysis were conducted to assess the influence of key model parameters on the results. First, 1‐way sensitivity analyses were conducted by using the defined ranges or 95% CI of rates and probabilities, management effect, cost, and utility parameters, as described in Table 1. Second, a probabilistic sensitivity analysis with 10 000 samples was performed to assess the sensitivity of the results to simultaneous changes in multiple variables. Event rates, transition probabilities, and utilities were modeled using β or Weibull distributions, while costs were modeled using a γ distribution. Furthermore, the cost‐effectiveness acceptability curve was created to assess the likelihood of being cost‐effective at willingness‐to‐pay thresholds equivalent to 3 times the per‐capita GDP ($30 765 [212180.05 RMB]).

## Results

### Cost‐Effectiveness Estimation During the Study Period

The mean cost of intervention is $1003.52 (6921.08 RMB) in the management group during the study period (Table 2). The CVD‐related costs ($422.76 [2915.69 RMB] versus $503.97 [3475.78 RMB]) and productivity losses ($300.15 [2070.08 RMB] versus $589.29 [4064.22 RMB]) were lower for the management group compared with the control group, respectively. The QALY was 0.06 higher in the management group during the study period, with the ICER of the management group compared with the control group being $11196.15 (77217.61 RMB) per QALY, which was lower than the willingness‐to‐pay threshold of 3 times of GDP per capita ($30 765 [212180.05 RMB]).

**Table 2:** Cost‐Effectiveness Results During the Study Period

Outcomes	Management group	Control group	Changes
Total cost ($/￥)	$2598.45 (￥17920.99)	$1965.28 (￥13554.14)	$633.17 (￥4366.85)
Intervention‐related costs	$1003.52 (￥6921.08)	…	$1003.52 (￥6921.08)
Costs of CVD care*	$422.76 (￥2915.69)	$503.97 (￥3475.78)	$–81.21 (￥‐560.09)
Other health care costs*	$872.02 (￥6014.15)	$872.02 (￥6014.15)	0
Productivity losses*	$300.15 (￥2070.08)	$589.29 (￥4064.22)	$–289.14 (￥‐1994.14)
QALY	6.95	6.89	0.06
ICER ($/QALY) (￥/QALY)	$11196.15 (￥77217.61)		

CVD indicates cardiovascular disease; ICER, incremental cost‐effectiveness ratio; and QALY quality‐adjusted life‐year.

* Costs of CVD care included costs for myocardial infarction and stroke. Other health care costs included costs for hypertension screening or checkups and costs for antihypertensive drugs.

### Lifetime Cost‐Effectiveness Estimation

The results of lifetime analyses are presented in Table 3. Total lifetime cost remained significantly higher ($1638.64; 11301.37 RMB) for the management group ($5772.24 versus $4133.59). The management group demonstrated a superiority of 0.88 QALY or 0.92 life‐year over the control group. The ICER for workplace‐based management, compared with routine care, was $1855.47 (12796.81 RMB) per QALY gained and $1780.27 (12278.17 RMB) per life‐year gained, respectively. Scenario analyses show that, when the workplace‐based management was assumed to have no survival benefit beyond 10 years or have no benefit on productivity losses beyond 10 years, the ICERs for workplace‐based management were $14 783.57 (101959.33 RMB) and $2201.14 (15 180.82 RMB), respectively.

**Table 3:** Lifetime Cost‐Effectiveness Results for Base Case and Scenario Analyses

Outcomes	Costs ($/￥)			Effectiveness (QALY or life‐years)			ICER	% <3× GDP per capita/QALY
	Management group	Control group	Changes	Management group	Control group	Changes		
Base case analyses								
QALY	$5772.24 (￥39809.98)	$4133.59 (￥28508.54)	$1638.64 (￥11301.37)	17.46	16.58	0.88	$1855.47 (￥12796.81)	100%
Life‐year	$5772.24 (￥39809.98)	$4133.59 (￥28508.54)	$1638.64 (￥11301.37)	19.43	18.51	0.92	$1780.27 (￥12278.17)	100%
Scenario analyses								
Management has no survival benefit beyond year 10	$5826.88 (￥40186.83)	$4133.59 (￥28508.54)	$1693.28 (￥11678.21)	16.69	16.58	0.11	$14783.57 (￥101959.33)	99.69%
Management has no benefit on productivity losses beyond year 10	5442.67 (￥37537.01)	3498.75 (￥24130.18)	1943.92 (￥13406.83)	17.46	16.58	0.88	$2201.14 (￥15180.82)	100%

$1.00=6.8968RMB; The GDP per capita of China in 2019 was reported to be $10 255 from the World Bank report. GDP indicates gross domestic product; ICER, incremental cost‐effectiveness ratio; and QALY, quality‐adjusted life‐year.

Table S4 presents the outcomes of 1‐way sensitivity analyses, and Figure S2 displays a tornado diagram. Sensitivity analysis of varying model parameters, such as costs, transition probabilities, and health utilities, confirmed the cost‐effectiveness of the management program. The above parameters that had top effects on the cost‐effectiveness results were discount rate, productivity losses in the control group, and costs for intervention.

**Figure 2:**
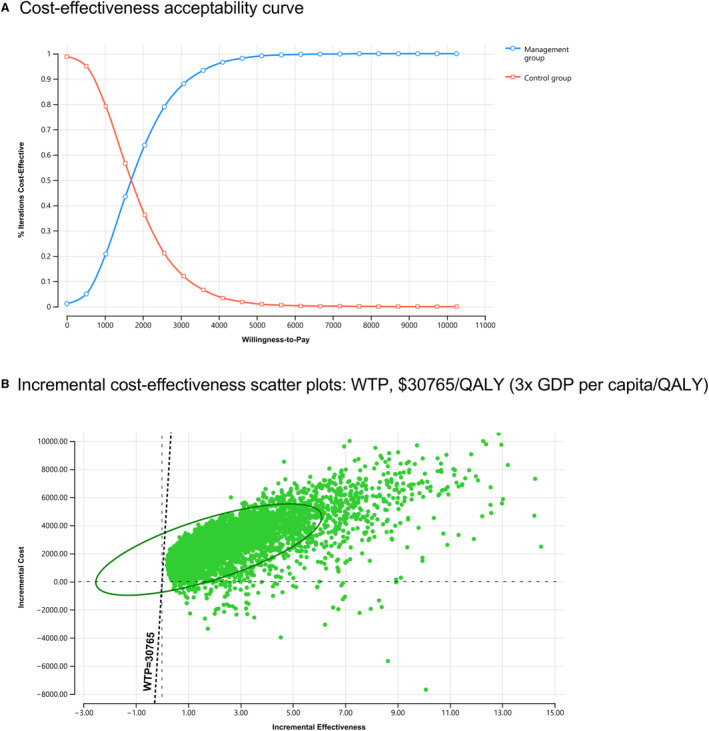
Cost‐effectiveness acceptability curve and incremental cost‐effectiveness scatter plots of the workplace‐based, multicomponent hypertension management program. **A**, Cost‐effectiveness acceptability curve. **B**, Incremental cost‐effectiveness scatter plots: WTP, $30 765/QALY (3× GDP per capita/QALY). GDP indicates gross domestic product; QALY, quality‐adjusted life year; and WTP, willingness‐to‐pay.

As shown in Figure 2, the management group had a 100% likelihood of being cost‐effective over the lifetime horizon, at a willingness‐to‐pay threshold of $30 765/QALY (3× GDP per capita/QALY).

## Discussion

To the best of our knowledge, this study represents the first investigation into the cost‐effectiveness of a workplace‐oriented hypertension management initiative overseen by community health workers in Chinese occupational populations, including both manual and mental labor workers. The lifetime model‐based economic analysis indicated that a workplace‐based hypertension management group was highly cost‐effective for male employees with hypertension aged 18 to 60 years compared with routine care.

Although BP reductions with different effect sizes have been found in previous studies.^6^, ^7^, ^8^, ^9^, ^10^, ^11^ these benefits should be weighed against costs to determine whether scarce health care resources should be invested in workplace‐based hypertension management strategies. However, none of the aforementioned studies have assessed the cost‐effectiveness of a workplace‐centered approach to hypertension management, primarily due to insufficient cost or event data. Based on an ongoing hypertension management program in real‐world practice, our analyses showed that the program compared with routine care costs $1855 per QALY gained over a lifetime horizon. Our results may provide important evidence for policy makers and practitioners to develop cost‐effective workplace‐based strategies, which could improve employee health and boost productivity.

In addition to workplace‐based strategies, other hypertension control programs with multicomponent strategies have been tested to be cost‐effective: a community‐based intervention including BP monitoring, health education, and lifestyle counseling by community health workers or volunteers^26^, ^29^; a physician–pharmacist collaboration intervention focused on medication education and adherence improvement, plus lifestyle modifications^30^; a telemonitoring system for home BP measurements coupled with pharmacist‐led case management^31^; and government initiatives aimed at promoting a decrease in salt consumption.^32^ Although acknowledging variations in practice settings and challenges of directly comparing costs and effectiveness between our study and previous programs, all studies have consistently shown that some strategies, such as regular BP monitoring, maintaining a healthy diet, encouraging treatment, and improving adherence, could be cost‐effective ways in achieving long‐term BP control and lowering the burden of hypertension.

Our findings have several public health implications. As recommended by the American Heart Association,^12^ the workplace is an important setting for preventing risk factors and reducing adverse outcomes for CVD. The prevalence of hypertension and modifiable risk factors for hypertension are prevalent in the workplace,^33^, ^34^ creating a large target population that stands to benefit from continuous engagement in hypertension management programs. In addition, the study population had comparable traits to present‐day working populations in China. For instance, participants underwent routine health assessments, had access to health education, and were enrolled in medical insurance. Thus, our findings support the feasibility, acceptability, and generalizability of the management program to be scaled up.

The study's strength lies in using a well‐established ongoing management program with a relatively long follow‐up and a large sample size to derive relevant parameters. In addition, enrollment in a hypertension management program is voluntary; therefore, the results may represent real‐world cost‐effectiveness and are more likely to be applied to other worksite settings. There were some limitations. First, the study was undertaken in a “real‐world” practice with nonrandom allocation of participants. Although we achieved balanced characteristics between the 2 groups using statistical methods, we cannot entirely rule out the possibility of unmeasured confounders and selection bias. Additionally, there may be a healthy participant effect or motivational bias, as individuals in the management group may inherently be more focused on their health compared with the control group,^35^ which calls for a more cautious interpretation of the findings. Second, our model simulated only a restricted range of health conditions and associated costs, potentially leading to an underestimation of the benefits or harms associated with the management program. Third, due to the lack of robust data on medication adherence and compliance with management activities, the potential impact on our results warrants further consideration. Fourth, the large benefits of the hypertension management program should be interpreted as the cost‐effectiveness of a multicomponent strategy, whereas the cost‐effectiveness of each individual component, such as providing free antihypertensive medications, cannot be obtained. Fifth, participants discontinued participation in workplace‐based hypertension management for semimonthly BP measurements at the time of retirement. Still, they were asked to visit community health centers to monitor their BP levels and received face‐to‐face health education at least once every 3 months, and will be asked to increase the frequency of BP monitoring if they have poorly controlled BP.^36^ In addition, the scenario analysis by assuming the management has no benefit after 10 years still showed similar results. Therefore, since most of the management measures are similar before and after retirement, we hypothesized that workplace‐based hypertension management might be potentially applicable to the retired population, and thus the lifetime benefits and costs were simulated. Sixth, the study was exclusively carried out among male individuals with hypertension. In addition, all participants were required to enroll in health insurance and covered by a uniform reimbursement policy.^5^ Thus, our results might not be directly extrapolated to the female working population or workers in other countries because of marked differences in treatment costs, event transition probabilities, and health insurance coverage among different populations. Finally, further studies are essential to validate our findings and establish a consensus, as relying on a single study for economic analysis remains insufficient and offers only a partial basis for decision making.

## Conclusions

The present study from the real‐world experience suggests that, over a lifetime horizon, a workplace‐based, multicomponent intervention of integrating a general workplace wellness program and hypertension management with regular BP measures was cost‐effective compared with routine care for men with hypertension without CVD. Future research should continue to validate the cost‐effectiveness of the workplace‐based strategy in diverse working populations in China and other regions.

## Sources of Funding

Dr Pan was supported by grants from the National Key Research and Development Program of China (2022YFC3600600) and the National Natural Science Foundation of China (82192902, 82021005, and 81930124). The funders had no role in the design and conduct of the study; collection, management, analysis, and interpretation of the data; preparation, review, or approval of the manuscript; and decision to submit the manuscript for publication.

## Disclosures

None.

## Supporting information

Data S1
Tables S1–S4
Figures S1–S2
