# Supplementary material for: Cost‐Effectiveness of a Workplace‐Based Hypertension Management Program in Real‐World Practice in the Kailuan Study
Source: J Am Heart Assoc. 2024 Apr 2;13(8):e031578. doi: 10.1161/JAHA.123.031578 (PMC11262526; doi:10.1161/JAHA.123.031578)
Supplement: Supplementary file 1 — Data S1 Tables S1–S4 Figures S1–S2 [file JAH3-13-e031578-s001.pdf]

SUPPLEMENTAL MATERIAL

Data S1.

Supplemental Methods

Propensity-matched analysis

Because the program enrollment was voluntary and not randomized, substantial differences in baseline characteristics existed between patients in the program and those in the control; therefore a propensity-matched analysis was applied. We included as many variables as possible in the propensity score model to reduce treatment assignment bias, and the model were performed separately for each examination cycle. The following baseline variables were included: sociodemographic factors (age, educational level, and occupational type), measured systolic BP and diastolic BP, body mass index [BMI], lifestyle factors (physical activity, drinking habits, smoking status, and salt intake), antihypertensive medications use, BP control status, elevated low-density lipoprotein cholesterol (LDL-C) levels, reduced high-density lipoprotein cholesterol (HDL-C) levels, elevated triglyceride levels, medical comorbidity (diabetes, stroke, MI, cancer, and impaired renal function), and family history of diseases (hypertension, stroke, MI, and diabetes). Then, we calculated a propensity score for each patient, regardless of statistical significance of the independent variables in the model. Finally, patients were matched one-to-one without replacement using a nearest-neighbor approach with caliper width of 0.20 standard deviations (SDs).

Table S1. Baseline characteristics of study participants.

Variables	Management group (N=6120)	Control group (N=6120)
Age, mean (SD), y	45.18 (6.40)	45.65 (7.92)
BMI, mean (SD), kg/m^2	26.15 (3.41)	26.06 (3.42)
Systolic BP, mean (SD), mm Hg	142.7 (16.1)	142.7 (18.3)
Diastolic BP, mean (SD), mm Hg	94.1 (10.5)	94.1 (11.2)
Education		
≤Middle school	4588 (75.0)	4484 (73.3)
High school	1145 (18.7)	1137 (18.6)
≥College	387 (6.3)	499 (8.1)
Self-reported manual labor work	5703 (93.2)	5608 (91.6)
Antihypertensive medication	994 (16.2)	946 (15.5)
Controlled blood pressure [*]	1104 (18.0)	1084 (17.7)
Physically active [†]	626 (10.2)	534 (8.7)
Current smoker	3552 (58.0)	3602 (58.9)
Current drinker	3696 (60.4)	3834 (62.7)
Light salt [‡]	1288 (21.1)	1203 (19.7)
Reported MI	67 (1.1)	61 (1.0)
Reported stroke	99 (1.6)	90 (1.5)
Diabetes	843 (13.8)	882 (14.4)
Cancer	17 (0.3)	15 (0.2)
Impaired renal function [§]	871 (14.2)	858 (14.0)
Elevated LDL-C [‖]	243 (4.0)	233 (3.8)
Reduced HDL-C [‖]	674 (11.0)	676 (11.0)
Elevated triglyceride [‖]	1821 (29.8)	1873 (30.6)
Family history of hypertension	1190 (19.4)	1312 (21.4)
Family history of MI	138 (2.3)	163 (2.7)
Family history of stroke	324 (5.3)	350 (5.7)
Family history of diabetes	387 (6.3)	414 (6.8)

BP indicates blood pressure; BMI, body mass index; HDL-C, high-density lipoprotein cholesterol; LDL-C, low-density lipoprotein cholesterol; MI, myocardial infarction.

[*] Controlled blood pressure was defined as a systolic blood pressure <140 mm Hg and a diastolic blood pressure <90 mm Hg.

[†] Being physically active was defined as moderate or vigorous physical activity for ≥80 minutes per week.

[‡] Light salt was defined as <6 g/day according to the standard salt spoon in China.

[§] Impaired renal function was defined as the presence of albuminuria or estimated glomerular filtration rate <60 (ml/min/1.73 m^2).

[||] Elevated LDL-C was defined as LDL-C ≥4.1 mmol/L (160 mg/dL), reduced HDL-C was defined as HDL-C <1.04 mmol/L (40 mg/dL), elevated triglycerides was defined as triglycerides ≥2.3 mmol/L (200 mg/dL).

Table S2. Early retirement for study participants.

Variables	Management group (n=6120)	Control group (n=6120)	*P* value
Early retirement because of illness or disability, N	68	91	0.04
Cumulative costs of early retirement during 10 years, $	2363231.64	4670680.552	-
Annual productivity loss per participant, $	38.61 (12.87-51.49) *	76.32 (25.44-101.76) *	-

US$1.00=6.8968RMB.

* A lower or higher estimate of productivity loss was calculated using a third or four-thirds of the average wage rate, respectively. The values inside the brackets represent ranges for sensitivity analyses.

Table S3. The average wage for each year in study participants.

Year	The average wage, $ *
2009	575.76
2010	645.72
2011	749.35
2012	828.30
2013	790.71
2014	775.62
2015	650.94
2016	691.44
2017	806.24
2018	964.59
2019	1016.40

* The costs were inflated to the 2019 price level using the average rate of inflation in China and converted to US dollars. US$1.00=6.8968RMB.

Table S4. Tornado text report (ICER), management group versus control group.

Variable Name	Variable Low	Variable High	Low	High
Discount rate	0	0.08	511.22	4011.65
Cost for productivity in the control group	25.44	101.76	1380.03	2806.35
Cost for intervention	102.02	164.14	1224.11	2558.12
Cost for post-stroke	314.38	4465.29	1235.98	2099.79
HR reduction for non-CVD mortality	0.08	0.4	1462.71	2281.01
Cost for productivity in the management group	0	38.61	1123.35	1855.47
Utility of hypertension	0.79	0.95	1743.97	2159.17
Cost for stroke	1002.35	9431.43	1617.25	1937.95
Cost for antihypertensive drug	51.24	266.76	1807.29	2082.87
HR reduction for stroke risk	0.01	0.26	1749.14	1957.28
Utility of post-stroke	0.46	0.82	1784.90	1923.52
Utility of stroke	0.26	0.89	1829.71	1874.01
Long-term recurrent stroke	0.02	0.09	1839.16	1873.20
Cost for MI	1350.55	13570.66	1852.19	1864.53
Cost for post-MI	137.07	1233.69	1853.03	1862.54
Utility of post-MI	0.67	0.94	1854.50	1858.85
Cost for hypertension screening	26.64	29.44	1853.68	1857.26
Utility of MI	0.5	0.89	1855.23	1855.96
HR reduction for stroke mortality	0.17	0.44	1855.27	1855.72

CVD indicates cardiovascular disease; HR, hazard ratio; MI, myocardial infarction; and ICER, incremental cost-effectiveness ratio.

Figure S1. Participant flowchart.

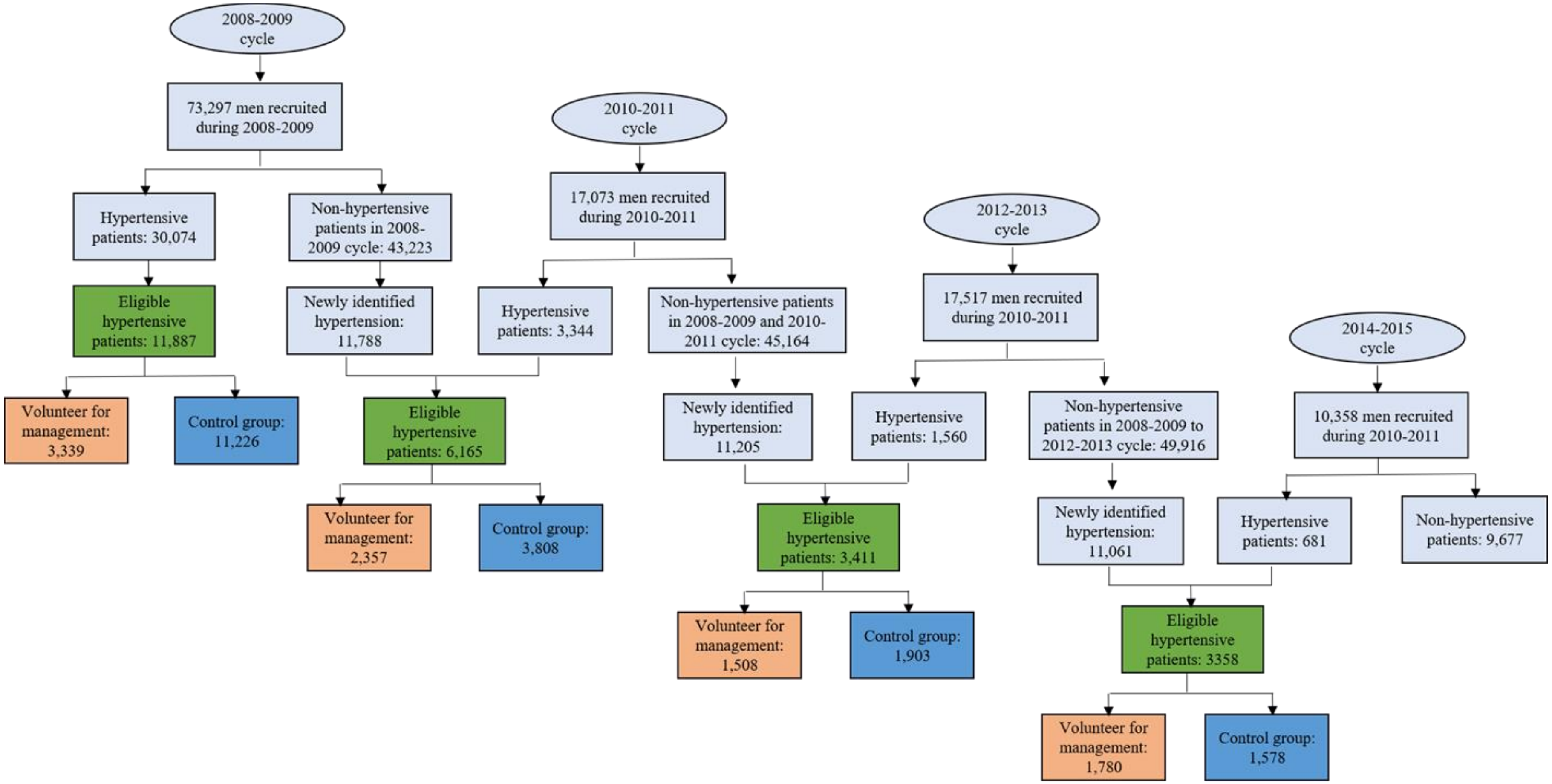

Figure S2. Tornado plot demonstrating the impact of varying each of the model parameters on the ICER for the management versus control groups.

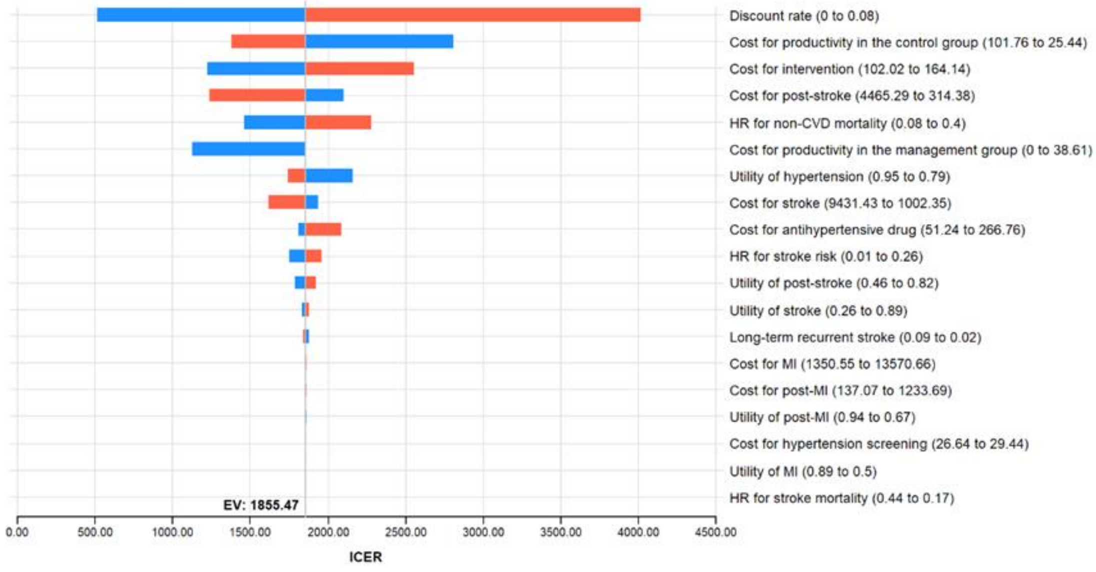

For the top parameter Capacity, the incremental value increases as the parameter increases with the blue bar section representing the parameter range from the low uncertainty value to the base case, while the red bar section represents the parameter range from the base case to the high uncertainty value.
